# Pre-arrest predictors of survival after resuscitation from out-of-hospital cardiac arrest in the elderly a systematic review

**DOI:** 10.1186/1471-2318-13-68

**Published:** 2013-07-03

**Authors:** Esther MM van de Glind, Barbara C van Munster, Fleur T van de Wetering, Johannes JM van Delden, Rob JPM Scholten, Lotty Hooft

**Affiliations:** 1 Department of Internal Medicine, Section of Geriatrics, Academic Medical Center, Amsterdam, the Netherlands; 2 Dutch Cochrane Centre, Academic Medical Center, University of Amsterdam, Amsterdam, the Netherlands; 3 Gelre Hospitals, Department of Geriatric Medicine, Apeldoorn, the Netherlands; 4 Julius Center for Health Sciences and Primary Care, University Medical Center, Utrecht, the Netherlands

**Keywords:** Cardiopulmonary Resuscitation, Geriatrics, Out-of-hospital, Prognostic factors, Systematic review

## Abstract

**Background:**

To enable older people to make decisions about the appropriateness of cardiopulmonary resuscitation (CPR), information is needed about the predictive value of pre-arrest factors such as comorbidity, functional and cognitive status on survival and quality of life of survivors. We systematically reviewed the literature to identify pre-arrest predictors for survival, quality of life and functional outcomes after out-of-hospital (OHC) CPR in the elderly.

**Methods:**

We searched MEDLINE (through May 2011) and included studies that described adults aged 70 years and over needing CPR after OHC cardiac arrest. Prognostic factors associated with survival to discharge and quality of life of survivors were extracted. Two authors independently appraised the quality of each of the included studies. When possible a meta-analysis of odd’s ratios was performed.

**Results:**

Twenty-three studies were included (n = 44,582). There was substantial clinical and statistical heterogeneity and reporting was often inadequate. The pooled survival to discharge in patients >70 years was 4.1% (95% CI 3.0-5.6%). Several studies showed that increasing age was significantly associated with worse survival, but the predictive value of comorbidity was investigated in only one study. In another study, nursing home residency was independently associated with decreased chances of survival. Only a few small studies showed that age is negatively associated with a good quality of life of survivors. We were unable to perform a meta-analysis of possible predictors due to a wide variety in reporting and statistical methods.

**Conclusions:**

Although older patients have a lower chance of survival after CPR in univariate analysis (i.e. 4.1%), older age alone does not seem to be a good criterion for denying patients CPR. Evidence for the predictive value of comorbidities and for the predictive value of age on quality of life of survivors is scarce. Future studies should use uniform methods for reporting data and pre-arrest factors to increase the available evidence about pre arrest factors on the chance of survival. Furthermore, patient-specific outcomes such as quality of life and post-arrest cognitive function should be investigated too.

## Background

Cardiopulmonary resuscitation (CPR), which was developed in the 1950s [1], is a treatment for cardiac arrest, which is a potentially lethal condition. Unfortunately, the success rates for CPR are poor. The percentage of patients who leave the hospital alive following the procedure varies from 0% to 20% and has not significantly improved in the last 30 years [2,3]. This might be caused by the increasing age of the population, longer EMS response time intervals attributable to urbanization and population growth and the declining incidence of ventricular fibrillation arrests [3].

With increasing age, the prevalence of morbidity and disability clearly increases, while perceived health status and physical well-being decrease [4-6]. The question arises whether CPR is appropriate for elderly patients who are multiply impaired and have limited life expectancy given their reduced likelihood of survival with a reasonable quality of life.

Many studies and reviews have reported on the chances of success. Sasson et al. studied the survival of out-of-hospital cardiopulmonary resuscitation and found that the success rate depends on arrest factors, such as witnessed arrest, provision of bystander CPR, shockable cardiac rhythm, time to arrival of ambulance and recovery of spontaneous circulation (ROSC) before hospital admission [3]. However, all or most of these factors are unknown when the decision about CPR is made. A recent meta-analysis by Ebell et al. [2] identified several pre-arrest predictors of failure to survive cardiopulmonary resuscitation for the in-hospital setting, although these factors were investigated in only few studies.

In spite of the wealth of literature, the exact effects of age and pre-arrest factors on survival remain unclear. Furthermore, it is unclear whether failure to survive in an out-of-hospital setting depends on age alone or on other pre-arrest factors such as cognitive impairment and comorbidity that are more prevalent at older ages [7,8].

In this systematic review, we aim to provide an overview of the current evidence on the association between pre-arrest factors and the probability of survival to discharge and beyond after out-of-hospital cardiac arrest (OHCA) and the quality of life of elderly (>70 years) survivors. This could inform the decision-making process about the desirability of cardiopulmonary resuscitation with evidence on the actual chances of survival in good health in patients with advanced age, comorbidity and/or nursing home residency.

## Methods

### Search strategy

We searched MEDLINE with an extensive search strategy to identify studies published between January 1980 and May 2011 that investigated prognostic factors for survival of out-of-hospital CPR (Additional file 1). In addition, we checked the reference lists of the selected studies to identify missing relevant articles and we used a multidisciplinary Dutch guideline about decision-making on resuscitation in older patients as an additional source of studies [9]. For this guideline, MEDLINE, Embase, Web of Science, CINAHL, Cochrane DSR, DARE and Cochrane Controlled Trial Register and DARE were searched to identify studies published between 1950 and 2008.

The root search was a combination of synonyms for cardiopulmonary resuscitation ([cardiopulmonary resuscitation] OR [CPR] OR [mouth to mouth]) and search terms for cardiopulmonary arrest ([heart arrest] OR [cardiac arrest] OR [cardiopulmonary arrest] OR [sudden death]) combined with outcomes ([quality of life] OR “cerebral recovery” [tiab] OR [functional impairment] OR “hospital discharge” [tiab]). All search terms were entered as free text words and as Medical SubHeadings (MeSH-terms). To limit the results to the geriatric population, we used a sensitive filter for geriatric medicine [10]. The search strategy is available through the authors.

### Study selection

First, one author (EvdG) selected studies based on the titles and abstracts. Then, two researchers (EvdG and FvdW) screened the full texts of the remaining articles more thoroughly. Disagreements were discussed with a third reviewer (LH). Only studies that were written in English were included.

For this review, we included studies that investigated patients who required CPR for a cardiopulmonary arrest in an out-of-hospital setting (including nursing homes). We defined cardiopulmonary arrest as the sudden cessation of spontaneous circulation and respiration leading to loss of consciousness and death when CPR is not provided. CPR was defined as the use of chest compressions and rescue breathing, with or without advanced life support, delivered according to the protocols that were applied in the study period.

We included studies in which the mean age of the participants was 70 years or more or in which different age groups were presented separately, including patients aged 70 years or more. Eligible studies assessed ‘age’ as a clinical predictor of survival or mortality after CPR, or as predictor for the quality of life of survivors, both univariably and multivariably. Studies also had to report ‘survival to discharge’ as a main outcome measure; studies that only reported ‘recovery of spontaneous circulation (ROSC)’ or ‘hospital admission’ were excluded. We excluded studies that described patients with loss of consciousness due to seizure, sole respiratory arrest or cardiopulmonary arrest due to trauma or drowning.

### Quality assessment

To assess the methodological quality of the included studies, we used a checklist based on the checklist developed by Hayden et al. [11]. This checklist assesses six domains of bias in a systematic review of prognosis studies (Table 1). Each item could score ‘low risk of bias’, ‘moderate risk of bias’, ‘high risk of bias’ or ‘unknown risk of bias’.

**Table 1:** Quality assessment of included studies

**Potential bias**	**Items to be considered for assessment of potential bias:**
Study participation	Low risk of bias was assessed if no patients group was excluded from the study cohort and when in- and exclusion criteria were adequately described.
	Moderate risk of bias was assessed if the sample was not adequately described for key characteristics. (age, sex, arrest characteristics).
	High risk of bias was assessed when both items were not adequately addressed.
Study attrition	Low risk of bias was assessed if there was no difference between eligible patients registered in a database and the number being analyzed. Also, there should have been no important differences between key characteristics and outcomes in participants who were analyzed the study and those who were not.
	Moderate risk of bias was assessed if loss to follow-up was described but was less than 20%.
	High risk of bias was assessed when loss to follow-up was not described or was >20%.
Prognostic factor measurement	Moderate risk of bias was assessed if at least the prognostic factor ‘age’ was taken into account.
	Low risk of bias was assessed when comorbidity and either functional dependence or comorbidity were reported.
	The prognostic factor measure and method should have been adequately valid and reliable to limit misclassification bias. The method and setting of measurement should have been the same for all study participants. When this was not the case, the risk of bias was assessed one step higher.
Outcome measurement	For the outcome ‘survival’, this item was not applicable.
	The outcomes ‘quality of life’ and ‘functional status’ of survivors were assessed separately. These outcomes should have been measured using a reliable and adequately valid method, in order to assess a low risk of bias.
Confounding measurement and account	Low risk of bias was assessed when was adjusted for all relevant confounders (shockable rhythm, witnessed arrest, provision of bystander CPR, interval to bystander or EMS CPR start). If was adjusted for only one or two factors, the risk of bias was assessed as moderately.
	Measurement of confounders should have been adequately valid and reliable, and method and setting of confounding measurement should be the same for all study participants.
	High risk of bias was assessed when no adjustment for confounders had taken place.
Analysis	Low risk of bias was assessed when there was sufficient presentation of data to assess the adequacy of the analysis. When only the significant factors were reported in the multivariate analyses, or when adjustment factors were not reported, the risk of bias was assessed higher.

Two researchers (EvdG and FvdW) independently performed the quality assessment. When necessary, disagreements were resolved through discussion with a third reviewer (LH).

### Data extraction

We used a standardized form to collect information on patients’ demographic and arrest characteristics. Furthermore, we extracted data on survival to discharge and beyond and on the quality of life, cognitive and functional status of survivors if reported. The reported arrest characteristics are all known to influence the outcome of cardiopulmonary resuscitation and can be considered as confounders for which analyses should be adjusted [3,12].

The outcomes ‘survival to discharge’ and’30-day survival’ were combined into short-term survival. Other outcome measures, such as long-term survival, quality of life and functional dependence of survivors, were reported separately.

### Analysis

For studies that exclusively included participants aged 70 years or above or reported data on a subgroup with this age and were sufficiently similar with respect to the participant and arrest factors, we calculated a pooled overall survival rate. We used an exact likelihood approach based on the binomial within-study distribution. This model allows for zero survivors in one or more studies [13]. Because we expected substantial heterogeneity in the reporting of quality of life and functional dependence, we did not pool these results. Nor did we perform a meta-analysis of the prognostic accuracy of the various individual prognostic factors, since we expected substantial heterogeneity between the primary studies, for example in the number and type of covariates that were studied and, more importantly, in the predictors that were included in a multivariable adjusted analyses (if done) [14,15].

## Results

### Identification of studies

**Figure 1:**
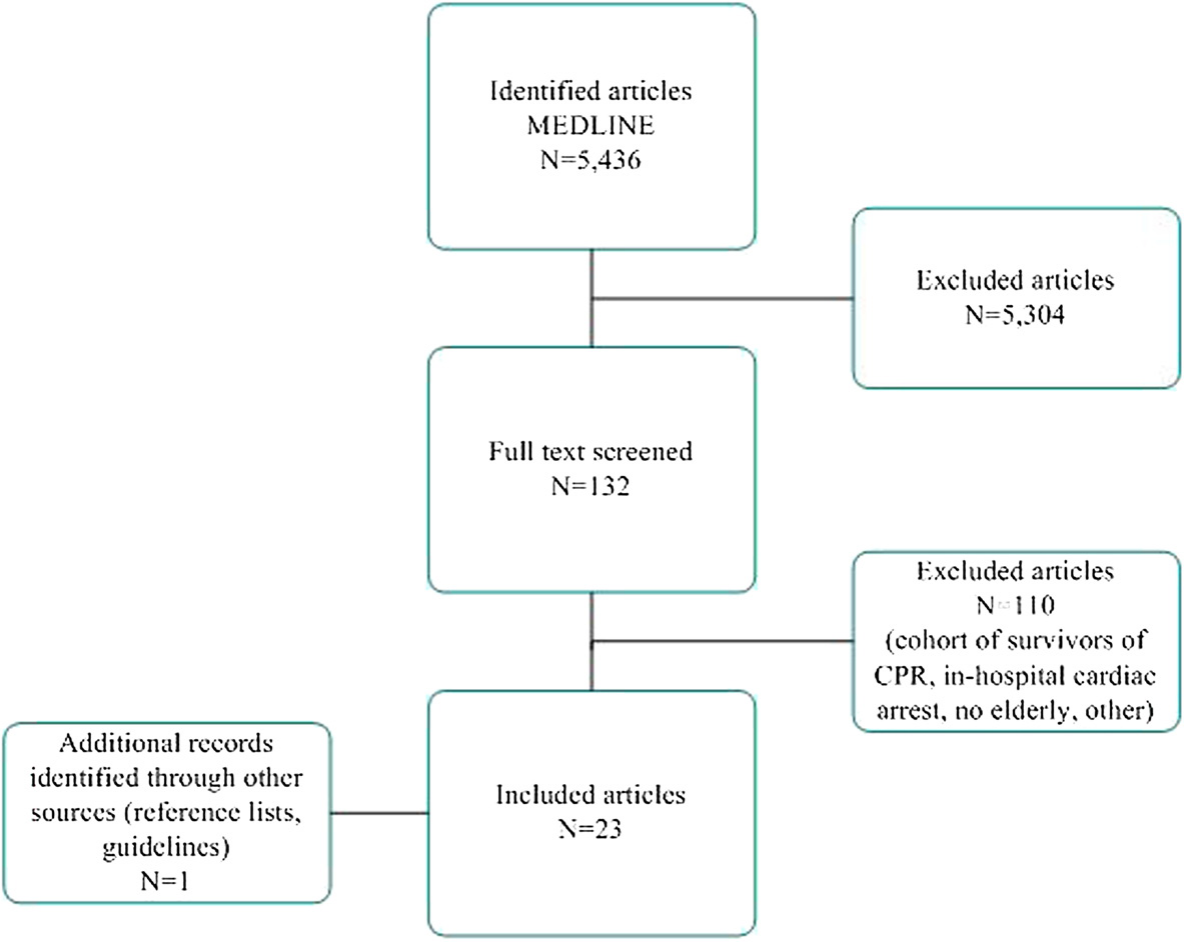
**Flowchart of selection of studies.** IHC = in-hospital cardiac arrest.

The search resulted in 5,436 articles (Figure 1). Of the 132 potentially relevant articles, 22 were included. In addition, one additional record was identified through other sources. The main reasons for exclusion were a mean age below 70 or no separate subgroup with participants >70 years and the examination of in-hospital CPR only. From the large Swedish cohort study by Herlitz, from which many reports were published, we included one key publication that met the inclusion criteria [16].

### Quality of the included studies

**Figure 2:**
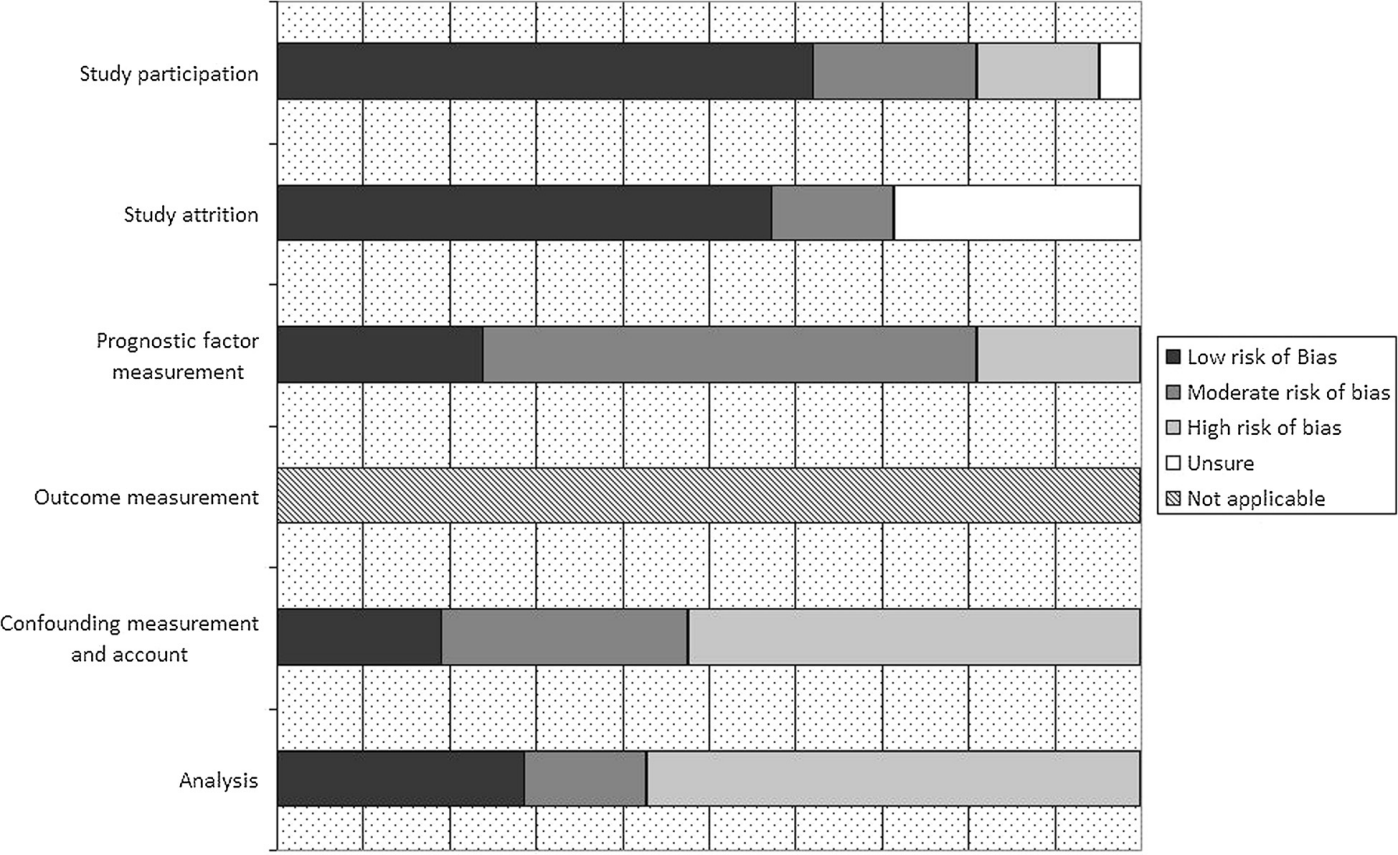
Quality assessment of included studies.

The majority of the studies scored a low-to-moderate risk of bias on the items of study participation, study attrition and prognostic factor measurement (Figure 2).

When a high risk of bias was assessed for the domain of study participation, it was because important baseline characteristics such as comorbidity or age were missing. In addition, some studies addressed a specific patient group that did not match the current research question. For example, some studies only described witnessed arrests or only included patients who were admitted alive [17,18]. The reasons for not reporting on the entire cohort of resuscitated patients in the analysis were not always listed. Therefore, it was not clear whether there were differences between the participants who were analyzed and those who were not, which could have introduced bias on the item of study attrition.

On the item of prognostic factor measurement, most of the studies were assessed as having a moderate risk of bias. Typically, the prognostic factor ‘age’ was described; however, other factors, such as co-morbidity or functional dependence, were often not reported. In these cases, the score on this domain was ‘moderate risk of bias’ at best.

In all cases the outcomes quality of life and functional and cognitive status of survivors were measured adequately and reliable, and therefore we assessed no risk on misclassification bias on this item.

In only a few cases, the items of confounding and analysis could be assessed as low risk of bias, because adjustment for response time, percentage bystander CPR and type of rhythm was generally poorly controlled. Furthermore, when a multivariate analysis was performed, only the variables that were statistically significantly associated with the outcome in the univariate analysis were typically presented, explaining the low score on the ‘analysis’ item.

### Characteristics of the included studies

Additional file 2: Table S2 shows the characteristics of the 23 included studies. The total number of included patients was 44,582, with an age range of 33–99 years. Of the studies, four exclusively included elderly patients [17,19-21]. In five of the studies, the mean age of the included patients was 70 years or above [22-26]. Fourteen studies provided a subgroup of elderly patients. For these studies, only the proportion survival in the oldest group is presented in Additional file 2: Table S2 [16,18,27-38].

Thirteen studies were performed in the USA, and eight in Europe. The study populations and registered pre-arrest characteristics varied across studies. All studies were retrospective cohort studies or chart reviews, with the exception of Ghusn [17], which was a case control study. All but one study [38] reported at least survival to discharge; seven reported long term outcomes as well [23,25,27,28,37,38].

## Findings of the included studies

### Survival

**Figure 3:**
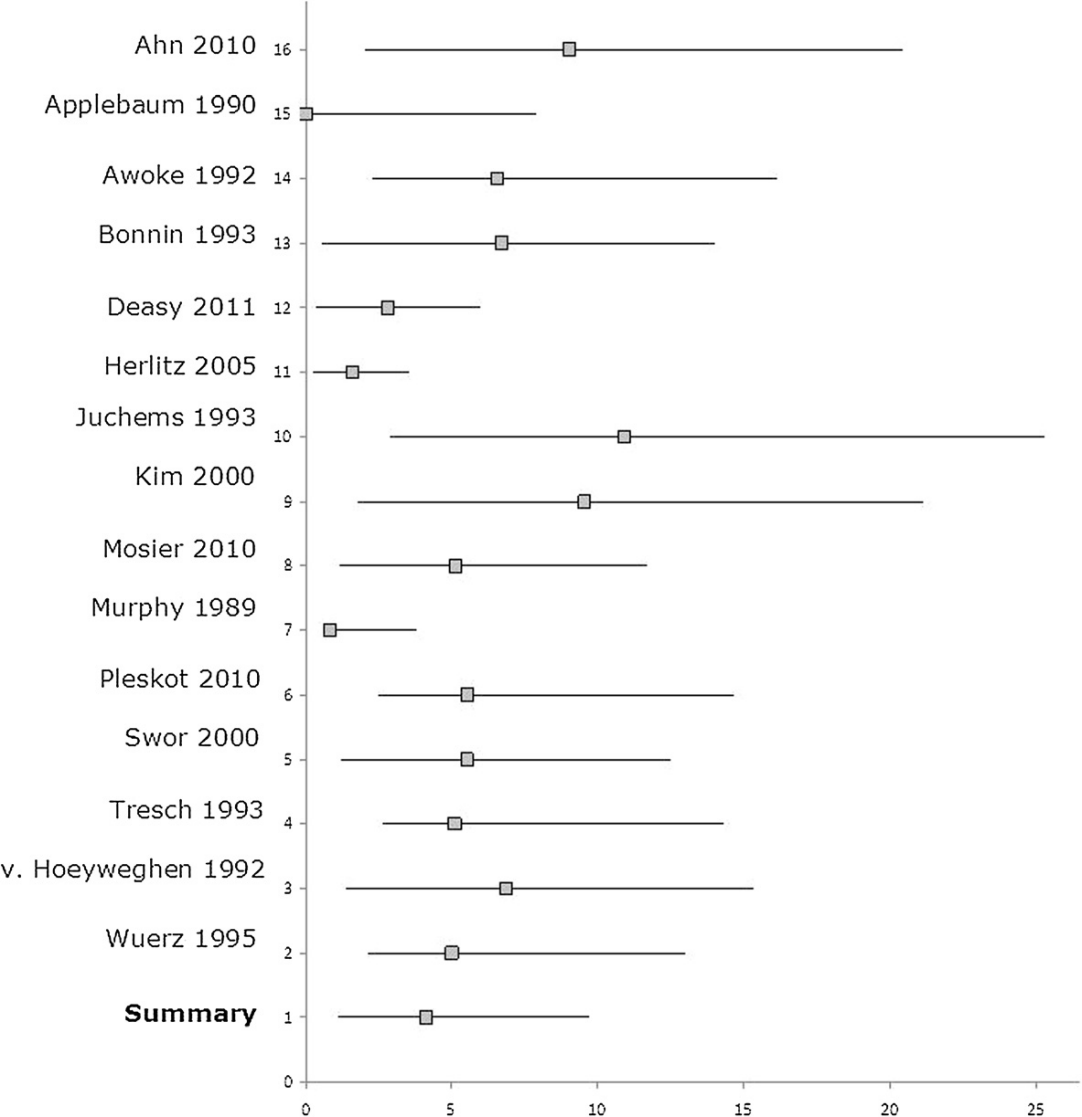
Pooled survival to discharge for patients aged 70 years and over after out of hospital cardiopulmonary resuscitation (%).

Fourteen studies were sufficiently clinically homogeneous to perform a meta-analysis for survival (Figure 3). For patients aged 70 years or older, the pooled overall survival to discharge was 4.1% (95% confidence interval (CI) 3.0-5.6%; range 0–9.0%) (Table 2). This was lower than the general survival, as reported by Sasson et al. (pooled survival 7.6% (95% CI 6.7-8.4%) [3].

**Table 2:** Reported odd’s ratio’s (OR) of included studies for survival after CPR

	**Prognostic factor**	**Crude ORs (95% CI)**	**OR in multivariate analysis (95% CI)**	**Factors included in multivariate analysis**
Applebaum 1990 [19]	Nursing home residency	0.14 (0.04-0.61)	NR	Not applicable
Ahn 2010 [22]	Age 15–64 y	1.0	1.0	Gender, age, location, witness, initial rhythm, elapsed time interval before start BLS^1^ and ALS^2^, level of EMS^3^ provider (basic or intermediate).
	Age ≥ 65 y	0.54 (0.44-0.65)	0.50 (0.41-0.62)	
	Gender (male)	1.57 (1.29-1.92)	1.14 (0.93-1.42)	
Deasy 2011 [30] (Non-shockable rhythms)	Age 65–69 y	1.0	1.0	Witnessed arrest, year in which arrest took place, sex, provision of bystander CPR, EMS response time, location of arrest.
	Age 70–74 y	0.87 (0.69-1.09)	0.93 (0.73-1.19)	
	Age 75–59 y	0.84 (0.68-1.05)	0.88 (0.69-1.11)	
	Age 80–84 y	0.78 (0.62-0.97)	0.86 (0.67-1.09)	
	Age 85–89 y	0.61 (0.48-0.79)	0.65 (0.49-0.85)	
	Age 90–94 y	0.42 (0.30-0.60)	0.45 (0.31-0.65)	
	Age 95–99 y	0.20 (0.08-0.50)	0.21 (0.08-0.52)	
Deasy 2011 [30] (Shockable rhythms)	Age 65–69 y	1.0	1.0	Witnessed arrest, year in which arrest took place, sex, provision of bystander CPR, EMS response time, location of arrest.
	Age 70–74 y	1.17 (0.92-1.49)	1.25 (0.97-1.61)	
	Age 75–59 y	1.24 (0.98-1.58)	1.29 (1.00-1.65)	
	Age 80–84 y	0.92 (0.71-1.19)	0.87 (0.66-1.15)	
	Age 85–89 y	0.85 (0.62-1.18)	0.82 (0.59-1.15)	
	Age 90–94 y	0.75 (0.45-1.25)	0.72 (0.42-1.24)	
	Age 95–99 y	0.12 (0.01-0.93)	0.11 (0.01-0.87)	
Fabbri 2006 [27]^4^	Age >74 y vs. <74	0.39 (0.21-0.71)	0.41 (0.87-0.93)	Initial rhythm, sex, age, comorbidity (history of diabetes, hypertension, myocardial infarction), seasonality, day-week, day-times, urban setting, home location, response times.
	Gender (male)	2.21 (1.11-4.41)	3.5 (1.18-10.36)	
	Heart failure^41^	0.04 (0.03-0.31)	0.37 (0.14-0.99)	
	Cardiovascular disorder	0.28 (0.11-0.72)	0.40 (0.16-1.00)	
		0.38 (0.17-0.86)	0.34 (0.14-0.83)	
	Hypertension	0.36 (0.16-0.82)	0.70 (0.58-0.85)	
	Diabetes mellitus			
Herlitz 2005 [16]	Age > 73 y vs. < 73 y	0.53 (0.46-0.62)	0.63 (0.50-0.71)	Witnessed arrest, initial rhythm, provision of bystander CPR, ALS response interval, age, sex.
		1.14 (0.97-1.33)	NR	
	Gender (male)			
Iwami 2006 [38]	Nursing home	0.96 (0.39-2.4)	NR	
Kim 2000 [32]	Age (per decade)	NR	0.92 (0.85-0.99)	Witnessed arrest, initial rhythm, sex, age, provision of bystander CPR, location of arrest.
	Gender (male)	NR	1.03 (1.32-0.77)	
Mosier 2010 [33]	Age (per decade)	NR	0.79 (0.67-0.93)	Witnessed arrest, VF^5^, agonal respirations, EMS response time, age.
Swor 2000 [34]	Age 50–59 y	1.0	1.0	Witnessed arrest, VF, provision of bystander CPR, ALS response interval <9 min.
	Age 60–69 y	0.81 (0.52-1.26)	0.86 (0.52-1.42)	
	Age > 70-79 y	0.70 (0.44-1.10)	0.83 (0.50-1.37)	
	Age > 80 y	0.31 (0.17-0.57)	0.40 (0.20-0.82)	

^1^Basic life support.

^2^Advanced life support.

^3^Emergency Medical Service.

^4^Fabbri: favourable outcome at discharge (= survival with an overall Performance Category 1–2).

^5^Ventricular fibrillation.

There was substantial clinical and statistical heterogeneity and reporting of statistical methods was often inadequate. Performing a meta-analysis of odd ratio’s (ORs) was impossible due to a wide variety in the statistical methods used such as the adjustment factors and the statistical models. Table 2 shows that the chance of survival significantly decreased as age increased, both in univariate [16,22,27,30,32-34,38] and multivariate analyses [16,22,27,30,32-34]. In the multivariate analyses, most studies included only arrest factors, such as ‘witnessed arrest’, ‘bystander arrest’ and ‘shockable rhythm’ in the model; thus, these studies do not clarify the influence of pre-arrest comorbidity and functional status. Only the study of Fabbri [27] analyzed the effect of pre-arrest co-morbidities on the chance of survival (see Table 2). However, this study did not adjust for arrest factors and only examined witnessed arrests. Of the 23 included studies, six studies investigated the predictive value of nursing home residency for decreased survival to discharge [17,19,20,30,32,38] (Table 3). For this group, the absolute survival chances were low and ranged from 0–5.1%. One study showed that nursing home residency was significantly associated with a lower chance of survival to discharge (OR 0.14) [19], whereas Deasy et al. presented a significant OR of 0.26 that was adjusted for arrest factors [30]. Although there were limited studies these data show that the chance of survival for this group is lower.

**Table 3:** Reported odd’s ratio’s (OR) for nursing home residence of included studies for survival after CPR

	**Prognostic factor**	**Crude ORs (95% CI)**	**OR in multivariate analysis (95% CI)**	**Factors included in multivariate analysis**	**Outcome**
Iwami 2006 [38]	Nursing home (witnessed cases) vs. arrest in other place	0.96 (0.39-2.4)	NR	Not applicable	1 year survival
Applebaum 1990 [19]	Nursing home residents vs. matched cohort	0.14 (0.04-0.61)	NR	Not applicable	Surivival to discharge
Kim 2000 [32]	Arrest in nursing home	NR	0.61 (0.31-1.20)	Witnessed arrest, initial rhythm, sex, age, provision of bystander CPR, location of arrest	Survival to discharge
Awoke 1992 [20]	No comparison made: “no resident survived to discharge from the hospital”				Survival to discharge
Deasy 2011 [30]	Nursing home residency vs. arrest at home/public place/other (non shockable rhythms)	NR	0.26 (0.11-0.60)	Witnessed arrest, year in which arrest took place, sex, provision of bystander CPR, EMS response time, location of arrest.	Survival to hospital discharge
Ghusn 1995 [17]	Patients admitted alive: Nursing home residents vs.. matched cohort of older community residing persons	1.15 (0.55-2.45)	NR		Survival to discharge

### Quality of life, functional and cognitive status of survivors

Of the included studies, eleven reported on characteristics of survivors such as functional and cognitive status [18,21,23,27-29,32,33,37,38] (Additional file 2: Table S2). Quality of life of survivors was reported in only one study [25].

Two studies reported that 7.5% of the patients for whom resuscitation was attempted survived neurologically intact to one year; however, one of these studies only examined witnessed arrests [27], and the other did not specify this outcome for older patients [23]. In patients that did not regain and sustain vital signs in the field, only 0.6% survived to discharge neurologically intact [24]. Other studies that included only patients over 70 years showed that although the overall survival was low, the majority of the survivors displayed moderate to good cerebral performance [18,25,28,32,37]. The study of Pleskot et al. showed no difference between younger and older survivors in cerebral performance, but the number of survivors was insufficient to identify significant differences [28].

In the Horsted study, survivors rated two of the eight quality of life aspects of the SF-36 scale as significantly worse than the age-matched normative scores. However, no specification for age was made [25].

## Discussion

Our review showed that, in general, patients aged over 70 years had less chance of surviving to discharge after an out-of-hospital cardiac arrest (4.1% (95% CI 3.0-5.6%)) than the patients of all age groups reported in a previous review (pooled survival 7.6% (95% CI 6.7-8.4%) [3]. Furthermore, the factors of nursing home residency [19,20,30,32,38] and pre-arrest comorbidity [27] were associated with decreased chances of survival. It was striking that only one study investigated the predictive value of pre-arrest comorbidity [27]. Although the studies that reported on cognition, functional performance and quality of life of survivors were heterogeneous and not specifically concerned older people, the conclusion can be drawn that most of the survivors were in acceptable health.

Information on the quality of life of survivors was scarce in the included studies. In the literature, there are some other studies available that investigated the quality of life of a group of survivors. Two studies showed, that in all age groups, post-resuscitation patients rated their quality of life significantly lower than that of the general population [25,39], whereas others showed that survivors rated their quality of life the same as a matched cohort [32,40]. In one study, age above 80 years was independently negatively associated with a good quality of life compared to age- and sex-matched samples from a cohort study (OR 0.3 [95% CI 0.1-0.8]) [41]. Whether this lower quality of life would be a justification for a do-not-resuscitate order is hard to define and is dependent on patient’s preferences. Furthermore, because of the conflicting results, larger cohort studies are necessary to define the true effect of resuscitation on quality of life in post-resuscitation patients.

Although the available evidence on the effect of pre-arrest factors on survival is limited, it is important to accurately inform older people of their limited chances of survival following out-of-hospital CPR. Adams et al. showed that elderly patients’ beliefs regarding the chances of survival after CPR are overly optimistic. Similarly, physicians’ expectations of the likelihood of survival are not realistic [42]. However, older people understand prognostic information, and such information may alter their preferences with respect to resuscitation [43]. Decisions about CPR require the shared decision making of the physician and patient (or proxy). This kind of treatment decisions should be based on both scientific evidence and doctor’s and patient’s preferences.

Our review has several limitations most of which are related to the retrospective design and quality of the original studies [44]. Firstly, there was large heterogeneity in reported outcomes, variables and patient groups. Secondly, many studies did not report the number of cases for which CPR was not attempted. We assumed that this group was in poorer health than the group that experienced a CPR-attempt, thereby overestimating the chances of survival of the entire group. Notably, in the study by Deasy et al., the percentage of patients for whom resuscitation was not attempted increased with age [30]. Third, the reported survival percentages varied, which may be partially explained by the varied CPR protocols and access to emergency services across the studied cohorts. Furthermore, most authors only reported the factors that were significantly associated with survival, which resulted in a high risk of bias on this item.

Ebell et al. proposed guidelines for future research on survival after in-hospital CPR. We believe that most of these recommendations are valid for out-of-hospital CPR too [44]. Their most important recommendation is uniform reporting of predictor variables, in- and exclusion criteria, demographic data and definitions of cardiopulmonary arrest and resuscitation.

Although our aim was to provide older patients and their doctors with sufficient information about the chances of older people to survive resuscitation in good health, the available evidence appeared to be limited. From our data, it is not clear if age per se is a limiting factor, because most studies did not adjust for pre-arrest factors. Moreover, there was considerable statistical and clinical heterogeneity, because of which performing a meta-analysis of ORs was impossible.

Cohort studies of the predictors of survival of CPR with consistent reporting of the statistical methods and results of studies would facilitate the undertaking of meta-analysis. This would provide useful information for prognostication for elderly [14]. As quality of life and cognitive and functional status are even more important at older age than survival per se, these outcomes should be reported too in future studies. This would help both doctors and patients in decision-making about the desirability of cardiopulmonary resuscitation.

## Conclusion

Although older patients have a lower chance of survival after CPR in univariate analysis (i.e. 4.1%), older age alone does not seem to be a good criterion for denying patients CPR. Evidence for the predictive value of comorbidities and for the predictive value of age on quality of life of survivors is scarce. Nursing home residency [19,20,30,32,38] and pre-arrest comorbidity [27] were associated with decreased chances of survival. Future studies should use uniform methods for reporting data and pre-arrest factors to increase the available evidence about pre arrest factors on the chance of survival. Furthermore, patient-specific outcomes such as quality of life and post-arrest cognitive function should be investigated too.

## Competing interests

None of the authors have personal, financial or potential conflicts of interest. This research was conducted for fulfillment of doctoral studies for Esther van de Glind. Further support was provided by a grant from the Netherlands Organization for Health Research and Development (ZonMW).

## Authors’ contributions

EvdG performed the selection of studies, quality assessment, data-extraction, interpreted the data and drafted the initial manuscript. BvM was involved in the development of the protocol, the presentation of results, assisted in the interpretation of data and supervised the final manuscript. FvdW served as the second evaluator of the quality assessment and data-extraction. HvD critically reviewed the draft. RS was involved in the development of the protocol, the adaptation of the quality assessment tool, the statistical analysis and commented on the draft. LH conceived and designed the study, designed the quality assessment tool, supervised the process of quality assessment, data-extraction and data-interpretation and supervised preparing the manuscript. All authors read and approved the final manuscript.

## Pre-publication history

The pre-publication history for this paper can be accessed here:

http://www.biomedcentral.com/1471-2318/13/68/prepub

## Supplementary Material

Additional file 1 Search strategy for MEDLINE. Click here for file

Additional file 2: Table S2 Characteristics of included studies. Click here for file
