# Supplementary material for: Pre-arrest predictors of survival after resuscitation from out-of-hospital cardiac arrest in the elderly a systematic review
Source: BMC Geriatr. 2013 Jul 3;13:68. doi: 10.1186/1471-2318-13-68 (PMC3711933; doi:10.1186/1471-2318-13-68)
Supplement: Additional file 2: Table S2 — Characteristics of included studies. [file 1471-2318-13-68-S2.doc]

Additional file 2: Table S2 Characteristics of included studies

| **Author** | **Population** | **Patients (n)** | **Study period** | **Age (year, mean/**  **median (SD[[1]](#endnote-2)/range))** | | **Male (%)** | **Comorbidity (pre-arrest, %)** | | **Witnessed (%)** | **Shockable rhythm (%)** | **Time to start/SD (min)** | **Bystander CPR (%)** | **Survival to discharge/ 1- month (%)** | **Other (long term outcome, functional outcome, quality of life)** |
| --- | --- | --- | --- | --- | --- | --- | --- | --- | --- | --- | --- | --- | --- | --- |
| **Ahn 2010[1]** | Adults and children | 7962 | 2006-2007 | 15-64 y  >65y | 45.2%53.1% | 65.9 | NR |  | 46.7 | 5.5 | NR | NR | 2.0* |  |
| **Applebaum 1990[2]** | NH[[2]](#endnote-3) residents vs. non-residents | 697 | 1987 | 82 (65-101) |  | NR[[3]](#endnote-4) | NR |  | NR | NR | NR | NR | 9.0* | NR |
| **Awoke 1992[3]** | Long-term care residents | 45 | 1987-1990 | 75 (64-93) |  | 100 | ASCVD[[4]](#endnote-5)  COPD [[5]](#endnote-6)  HT [[6]](#endnote-7)  DM [[7]](#endnote-8)  CVA [[8]](#endnote-9)  Cancer | 36  33  20  20  13  4 | 38 | 27 | 5 min in 92% | NR | 0* | NR |
| **Bonnin 1989[4]** | Adult patients who do not regain and sustain vital signs in the field | 181 | 1986-1987 | 71 (18-99) |  | 61 | NR |  | 55 | 28 |  | 17 | 0.6 | 0.6% neurologically intact |
| **Bonnin 1993[5]** **Error: Reference source not found** | Adult patients | 367 | 1988-1989 | 78.5 (18-?) |  | 56 | NR |  | 30 | 32 | 11.3 (2-30) | 17 | 6.5* | NR |
| **Deasy 2011[6]** | Adult patients | 7625 | 2000-2009 | 70 (median) (52-80) |  | 65.5 | NR |  | 32 | 19 | 7 (6-9) | 24 | 6.7* | NR |
| **Fabbri 2006[7]** | Bystander witnessed CA | 244 | 1994-2004 | 73 median (IQR 64-73) |  | 66.2 | MI[[9]](#endnote-10)  DM  HT  CHF[[10]](#endnote-11) | 24  27  26  27 | 100 | 50 | 5 (4-7) | NR | 7.0* | 7.9% favorable outcome at 1 year |
| **Fischer 1997[8]** | Adult patients, witnessed arrest | 464 | 1989-1992 | > 70:  18-69: | 46%  54% | NR | NR |  | 62 | 45 | 16 | 16 | 15.9 | 7.5% of total were discharged without neurological deficit  11% of total survived to 1 year, 7.3% survived to 1 year without neurological deficit. |
| **Gushn 1995[9]** | Patients admitted alive:  Nursing home residents vs. | 114 | 1986-1991 | 80.8 (±5.6) |  | 37.5 | ASCVD  COPD  CVA  Cancer  Alzheimer  DM  HT  CRF[[11]](#endnote-12) | 25  22  10  11  1  17  3  2 | 65 | 17 | 9.3 | 59 | 10.5* | NR |
| **Gushn 1995[9]** | matched cohort of older community residing persons | 228 |  | 80.7 (±5.6) |  | 37.7 | ASCVD  COPD  CVA  Cancer  Alzheimer  DM  HT  CRF | 55  6  10  11  3  11  10  1 | 77 | 32 | 9.4 | 34 | 9.2* | NR |
| **Herlitz 2005[10]** | All patients | 9067 | 1990-2005 | 71 (±12) |  | 75 | NR |  | 68 | 37 | 6 | 36 | 4.3* | NR |
| **Horsted 2007[11]** | Adult patients | 512 | 2002-2004 | 71 (34-91) Median) |  | 66.2 | NR |  | 78.5 | 34 | NR | 13.1 | 13.1 | Survival > 6 months:  0—50 y: 14.4%  51—70 y: 17.7%  71—90 y: 7.0%  > 91y: 0%  In 33 survivors, median MMSE was 29 (16-30), 6 had an MMSE < 24. SF-26: 2 out of 8 aspects were significantly worse. None of the summary scores were significantly different. |
| **Iwami 2006[12]** | Adult patients, not EMS witnessed | 7962 | 1998-2001 | 70.3 (15.5) |  | 59.4 | NR |  | 37.9 | 9.3 | NR | NR | NR | 1 year survival: 1.6%  Good neurological outcome: 0.9% |
| **Juchems 1993[13]** | Adult patients | 403 | 1981-1989 | >70 (range/mean ?) |  | NR | NR |  | NR | NR | NR | NR | 10.9 * | NR |
| **Kim 2000[14]** | Octogenarians and nonagenarians | 1300 | 1987-1998 | 80-89  >89 | 83%  93% | 56  30 | NR |  | 48  46 | 31  24 | 46  44 | 46  44 | 9.4*  4.4* | No differences in quality of life between survivors and a matched control group. |
| **Lombardi 1994[15]** | Adult patients | 2071 | 1990-1991 | 70 (median, IQR[[12]](#endnote-13) 60-79) |  | 59.3 | NR |  | 64 | 34 | 32 | 32 | 1.4 | NR |
| **Mosier 2010[16]** | Patients receiving cardiocerebral resuscitation (CCR) and ALS | 1209 | 2005-2008 | 66 (±15) |  | 67 | NR |  | 44 | 31 | 5.2 (2.3) | 44 | 5.1* | CCR group 96.6 % of 204 survivors had good neurologic outcome, in the ALS group 85% |
| **Murphy 1989[17]** | Patients ≥ 70 years including pts from a nursing home | 244 | 1978-1987 | 70-79  80-89  90-103 | 51.6%  39.8%  8.6% | 53.3 | NR |  | 49 | 25 | NR | NR | 0.8* | 47% of survivors little/no impairment; 10.5% severely impaired; 42% moderately impaired. |
| **Pleskot 2010[18]** | All patients | 253 | 2002-2004 | 77.1 (70-97) |  | 65 | HT  DM  Smoking  HC |  | 53  28  15  8 | 43 | 8 (5.5) | 41 | 5.5* | 1-y survival: 3%  At 30 days:  CPC 1-2: 4.7%  CPC 3-4: 0.8% |
| **Swor 2000[19]** | Adult patients | 1213 | 1989-1993 | 66.5 (±15.3, 19-112) |  | 63.3 | NR |  | 49 | 50 | 6.1 (3.9) | 20.1 | 5.5* | NR |
| **Tresch 1988[20]** | Pts who were successfully resuscitated and hospitalized. | 613 | NR | 33-55  56-64  65-69  70-74  75-79  80-99 | 18%  22%  14%  16%  14%  15% | 67.8 | NR |  | 54 | 44 | NR | <56: 76%, >56: 63% | 9.0* | 70% Functional status unchanged; 20% deteriorated, 10% improved. 40% of survivors survived >12 months (n=4) |
| **Tresch 1993[21]** | Nursing home patients | 196 | 1986-1989 | 78.5 (31-107) |  | 38 | ASCVD  HT  HF  Dementia  MI  DM  CVA  Pulmonary disease  Cancer | 52  43  42  38  26  24  21  17  10 | 49 | 20 | 0-15 min in 74% | NR | 5.1* | 70% Functional status unchanged; 20% deteriorated, 10% improved. 40% of survivors survived >12 months (n=4) |
| **Van Hoeyweghen[22] 1992** | Adult patients | 1153 | 1983-1987 | <40  40-69  70-79  >80 | 8%  50%  30%  12% | NR | Functionally normal  Disabled  Unconscious | 52  47  0.3 | 53 | NR | 8.8 (13.6) | NR | 6.9 (conscious at 14 days) * | NR |
| **Wuerz 1995[23]** | Patients >30 y with known initial rhythm | 320 | 1987-1991 | 75 ± 7 |  | NR | NR |  | 59 | NR | 9 (5) | 50  48 | 5.0* | NR |

Reference List

1. Ahn KO, Shin SD, Suh GJ, Cha WC, Song KJ, Kim SJ *et al*.: **Epidemiology and outcomes from non-traumatic out-of-hospital cardiac arrest in Korea: A nationwide observational study.** *Resuscitation* 2010, **81:** 974-981.

2. Applebaum GE, King JE, Finucane TE: **The outcome of CPR initiated in nursing homes.** *J Am Geriatr Soc* 1990, **38:** 197-200.

3. Awoke S, Mouton CP, Parrott M: **Outcomes of skilled cardiopulmonary resuscitation in a long-term-care facility: futile therapy?** *J Am Geriatr Soc* 1992, **40:** 593-595.

4. Bonnin MJ, Swor RA: **Outcomes in unsuccessful field resuscitation attempts.** *Ann Emerg Med* 1989, **18:** 507-512.

5. Bonnin MJ, Pepe PE, Clark PS, Jr.: **Survival in the elderly after out-of-hospital cardiac arrest.** *Crit Care Med* 1993, **21:** 1645-1651.

6. Deasy C, Bray JE, Smith K, Harriss LR, Bernard SA, Cameron P: **Out-of-hospital cardiac arrests in the older age groups in Melbourne, Australia.** *Resuscitation* 2011, **82:** 398-403.

7. Fabbri A, Marchesini G, Spada M, Iervese T, Dente M, Galvani M *et al*.: **Monitoring intervention programmes for out-of-hospital cardiac arrest in a mixed urban and rural setting.** *Resuscitation* 2006, **71:** 180-187.

8. Fischer M, Fischer NJ, Schuttler J: **One-year survival after out-of-hospital cardiac arrest in Bonn city: outcome report according to the 'Utstein style'.** *Resuscitation* 1997, **33:** 233-243.

9. Ghusn HF, Teasdale TA, Pepe PE, Ginger VF: **Older nursing home residents have a cardiac arrest survival rate similar to that of older persons living in the community.** *J Am Geriatr Soc* 1995, **43:** 520-527.

10. Herlitz J, Engdahl J, Svensson L, Angquist KA, Young M, Holmberg S: **Factors associated with an increased chance of survival among patients suffering from an out-of-hospital cardiac arrest in a national perspective in Sweden.** *Am Heart J* 2005, **149:** 61-66.

11. Horsted TI, Rasmussen LS, Meyhoff CS, Nielsen SL: **Long-term prognosis after out-of-hospital cardiac arrest.** *Resuscitation* 2007, **72:** 214-218.

12. Iwami T, Hiraide A, Nakanishi N, Hayashi Y, Nishiuchi T, Uejima T *et al*.: **Outcome and characteristics of out-of-hospital cardiac arrest according to location of arrest: A report from a large-scale, population-based study in Osaka, Japan.** *Resuscitation* 2006, **69:** 221-228.

13. Juchems R, Wahlig G, Frese W: **Influence of age on the survival rate of out-of-hospital and in-hospital resuscitation.** *Resuscitation* 1993, **26:** 23-29.

14. Kim C, Becker L, Eisenberg MS: **Out-of-hospital cardiac arrest in octogenarians and nonagenarians.** *Arch Intern Med* 2000, **160:** 3439-3443.

15. Lombardi G, Gallagher J, Gennis P: **Outcome of out-of-hospital cardiac arrest in New York City. The Pre-Hospital Arrest Survival Evaluation (PHASE) Study.** *JAMA* 1994, **271:** 678-683.

16. Mosier J, Itty A, Sanders A, Mohler J, Wendel C, Poulsen J *et al*.: **Cardiocerebral resuscitation is associated with improved survival and neurologic outcome from out-of-hospital cardiac arrest in elders.** *Acad Emerg Med* 2010, **17:** 269-275.

17. Murphy DJ, Murray AM, Robinson BE, Campion EW: **Outcomes of cardiopulmonary resuscitation in the elderly.** *Ann Intern Med* 1989, **111:** 199-205.

18. Pleskot M, Hazukova R, Stritecka H, Cermakova E: **Five-year survival of patients after out-of-hospital cardiac arrest depending on age.** *Arch Gerontol Geriatr* 2010.

19. Swor RA, Jackson RE, Tintinalli JE, Pirrallo RG: **Does advanced age matter in outcomes after out-of-hospital cardiac arrest in community-dwelling adults?** *Acad Emerg Med* 2000, **7:** 762-768.

20. Tresch DD, Thakur R, Hoffmann RG, Brooks HL: **Comparison of outcome of resuscitation of out-of-hospital cardiac arrest in persons younger and older than 70 years of age.** *Am J Cardiol* 1988, **61:** 1120-1122.

21. Tresch DD, Neahring JM, Duthie EH, Mark DH, Kartes SK, Aufderheide TP: **Outcomes of cardiopulmonary resuscitation in nursing homes: can we predict who will benefit?** *Am J Med* 1993, **95:** 123-130.

22. Van Hoeyweghen RJ, Bossaert LL, Mullie A, Martens P, Delooz HH, Buylaert WA *et al*.: **Survival after out-of-hospital cardiac arrest in elderly patients. Belgian Cerebral Resuscitation Study Group.** *Ann Emerg Med* 1992, **21:** 1179-1184.

23. Wuerz RC, Holliman CJ, Meador SA, Swope GE, Balogh R: **Effect of age on prehospital cardiac resuscitation outcome.** *Am J Emerg Med* 1995, **13:** 389-391.

1. * Reported results only for the subgroup of older patients.

   ? Standard deviation [↑](#endnote-ref-2)
2. Nursing home [↑](#endnote-ref-3)
3. Not reported [↑](#endnote-ref-4)
4. Atherosclerotic cardiovascular disease [↑](#endnote-ref-5)
5. Chronic Obstructive Pulmonary Disease [↑](#endnote-ref-6)
6. Hypertension [↑](#endnote-ref-7)
7. Diabetes Mellitus [↑](#endnote-ref-8)
8. Cerebrovascular accident [↑](#endnote-ref-9)
9. Myocardial infarction [↑](#endnote-ref-10)
10. Chronic heart failure [↑](#endnote-ref-11)
11. Chronic renal failure [↑](#endnote-ref-12)
12. Interquartile range [↑](#endnote-ref-13)
